# Stroke-like lesion and status epilepticus in a child with *NARS2*-related combined oxidative phosphorylation deficiency 24

**DOI:** 10.3389/fneur.2025.1731858

**Published:** 2025-12-05

**Authors:** Song Su, Wandong Hu, Yong Liu, Qiong Lang, Hongwei Zhang

**Affiliations:** 1 Department of Neurology, Children’s Hospital Affiliated to Shandong University, Jinan, Shandong, China; 2 Department of Neurology, Jinan Children’s Hospital, Jinan, Shandong, China; 3 Department of Child Health Care, Children’s Hospital Affiliated to Shandong University, Jinan, Shandong, China; 4 Department of Child Health Care, Jinan Children’s Hospital, Jinan, Shandong, China

**Keywords:** *NARS2*, COXPD24, infarction-like lesion, status epilepticus, mitochondrial disease

## Abstract

**Introduction:**

The *NARS2* gene encodes mitochondrial asparaginyl-tRNA synthetase, and biallelic pathogenic variants have been associated with combined oxidative phosphorylation deficiency 24 (COXPD24), an autosomal recessive mitochondrial disorder characterized by highly heterogeneous clinical manifestations. This study retrospectively analyzed the clinical and genetic findings of a Chinese infant presenting with status epilepticus and explored potential genotype–phenotype correlations.

**Methods:**

Clinical data, laboratory tests, neuroimaging, and disease course of the proband were reviewed. Whole-exome sequencing (WES) and copy-number variation (CNV) analysis were performed to identify causative variants in *NARS2*. Candidate variants were assessed by population database screening and literature review.

**Results:**

The proband, a 9-month-old girl, presented with status epilepticus, global developmental delay, increased muscle tone, elevated serum lactate and myocardial enzyme levels. Brain magnetic resonance imaging (MRI) revealed a focal cerebral lesion consistent with a metabolic or stroke-like infarction, as well as delayed myelination. WES identified compound heterozygous *NARS2* variants: a large exon 6–11 deletion and a novel missense variant c.467T>C (p.Leu156Ser), inherited in an autosomal recessive manner. Both variants were absent from public population databases and published literature. Notably, cerebral infarction has not been previously reported in *NARS2*-related disorders, suggesting a potential expansion of the clinical spectrum.

**Discussion:**

Review of previously reported *NARS2* variants indicates that both missense and loss-of-function mutations can lead to variable disease severity depending on residual enzyme activity. This case broadens the phenotypic and mutational spectrum of *NARS2*-associated COXPD24 and highlights the importance of evaluating large exon deletions and novel variants in infants with early-onset mitochondrial encephalopathy and epileptic manifestations.

## 1 Introduction

Combined oxidative phosphorylation deficiency type 24 (COXPD24, OMIM #616239) is an autosomal recessive mitochondrial disorder caused by biallelic pathogenic variants in the *NARS2* gene (chromosome 11q14.3), which encodes mitochondrial asparaginyl-tRNA synthetase (1, 2). This enzyme is a member of the mitochondrial aminoacyl-tRNA synthetasefamily and plays a crucial role in mitochondrial translation by catalyzing the conjugation of asparagine to its cognate mitochondrial tRNA. Proper aminoacylation is essential for the accurate synthesis of mitochondrial-encoded proteins that constitute key subunits of the oxidative phosphorylation (OXPHOS) system. Dysfunction of NARS2 disrupts this process, resulting in impaired assembly of respiratory chain complexes I, III, and IV, collapse of mitochondrial ATP production, and subsequent energy crisis in metabolically active tissues such as the brain, heart, and skeletal muscle (3, 4).

Since its first description by Simon et al. in 2015 in a family with sensorineural hearing loss, *NARS2*-related COXPD24 has been recognized as a clinically heterogeneous mitochondrial encephalomyopathy. The phenotypic spectrum ranges from isolated hearing impairment to severe multisystem neurodegenerative disease (5–7). Early-onset cases typically present during infancy with profound developmental delay, intractable epilepsy, muscular hypotonia or hypertonia, brain atrophy, and elevated serum lactate levels. Some patients exhibit Alpers-like features characterized by progressive encephalopathy with hepatic involvement. Approximately three-quarters of reported individuals develop sensorineural hearing loss, although neurological symptoms often precede auditory dysfunction, suggesting that neuronal energy failure may represent the earliest manifestation (8, 9).

The disease exhibits considerable genotypic and phenotypic variability, reflecting differences in the residual enzymatic activity of mutant NARS2 proteins. To date, 38 patients with *NARS2*-related COXPD24 have been reported worldwide, most harboring missense or nonsense variants. To our knowledge, no large exon deletions have been previously described. Here, we describe a Chinese infant diagnosed with COXPD24 carrying compound heterozygous *NARS2* variants: a large exon 6–11 deletion and a novel missense variant c.467 T > C (p. Leu156Ser), making this the first reported case involving a multi-exon deletion in *NARS2*.

The patient presented with status epilepticus, global developmental delay, elevated lactate and myocardial enzyme levels, and notably, stroke-like infarction on magnetic resonance imaging (MRI)—an unreported manifestation in *NARS2*-related disorders. This case expands both the phenotypic and mutational spectrum of *NARS2*-associated COXPD24 and provides new insights into the pathophysiological mechanisms underlying mitochondrial energy failure–related brain injury.

## 2 Patient information

### 2.1 Essential information

The proband was a 9-month-and-10-day-old girl admitted to the Department of Neurology, Children’s Hospital Affiliated to Shandong University, due to “recurrent convulsive seizures for 1 month.” At 8 months and 11 days of age, she developed episodes characterized by sudden limb jerking accompanied by gaze fixation, upward eye deviation, perioral cyanosis, and absence of urinary or fecal incontinence. Each episode lasted approximately 10 min and recurred about every hour, with oxygen desaturation during the attacks. The seizures were difficult to control even with sedative and symptomatic treatment. She had a history of intermittent limb tremors and was treated with oral levetiracetam after the onset of seizures, but the response was poor, with frequent recurrent seizures.

The patient exhibited global developmental delay: at 8 months of age she could not hold her head steadily, roll over, or sit independently. Visual tracking and auditory orientation were limited; she showed no response when her name was called and did not recognize her mother. Muscle tone was increased. She was the first child of healthy, non-consanguineous parents, born at full term by cesarean section without perinatal asphyxia. Physical examination revealed a body length of 71 cm, weight 7 kg, and head circumference 46 cm, all below the average for age. Developmental milestones were significantly delayed, with increased muscle tone, while no other abnormalities were observed.

### 2.2 Auxiliary examination

Laboratory evaluation revealed mildly elevated liver enzymes and repeatedly increased serum lactate and myocardial enzyme levels, while renal function, electrolytes, pancreatic enzymes, blood glucose, lipids, plasma ammonia, and homocysteine were within normal ranges. Specifically, serum lactate was 3.2 mmol/L (reference range: 0.50–2.22 mmol/L), alanine aminotransferase (ALT) 87 U/L (reference range: 8–71 U/L), aspartate aminotransferase (AST) 69 U/L (reference range: 21–80 U/L), creatine kinase (CK) 164 U/L (reference range: 28–287.6 U/L), and CK-MB 6.2 ng/mL (reference range: 0–5 ng/mL). No plasma pyruvate measurement was available; therefore, the lactate/pyruvate ratio could not be determined. Complete blood count, coagulation profile, and D-dimer levels were normal. Chromosomal karyotype analysis showed 46, XX. Plasma amino acid and acylcarnitine profiles, as well as urinary organic acid analysis, did not indicate any typical inborn error of metabolism.

**Figure 1:**
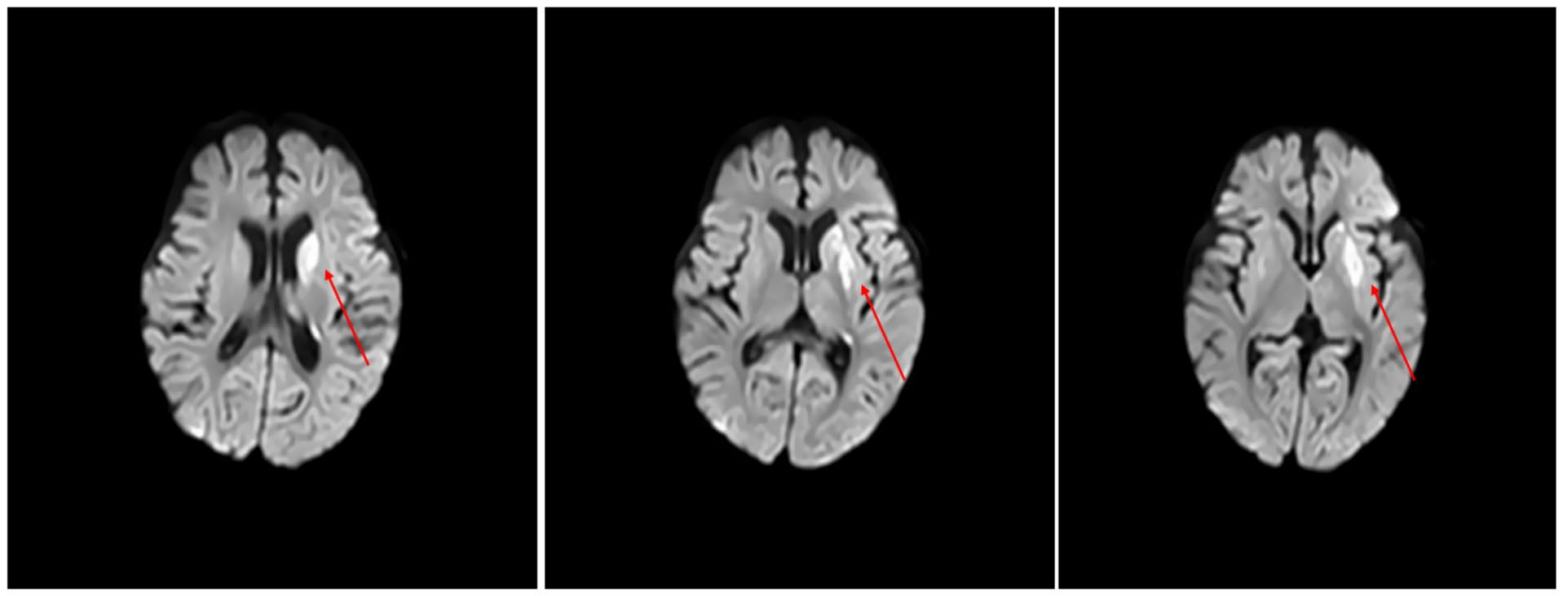
A brain MRI of the patient showing a markedly hyperintense lesion in the left cerebral hemisphere on DWI, indicating restricted diffusion consistent with infarction-like lesion.

Echocardiography indicated an interrupted echo in the mid-portion of the atrial septum, suggestive of a patent foramen ovale. Initial a brain MRI performed at admission demonstrated delayed myelination of the cerebral white matter compared with age-matched controls, without focal parenchymal abnormalities. During hospitalization, following an episode of status epilepticus and new-onset right-sided limb weakness, repeat MRI revealed newly developed strip- and patch-like lesions in the left frontal lobe, periventricular region, basal ganglia, thalamus, temporal lobe, and hippocampus. These lesions appeared hypointense on T1-weighted and hyperintense on T2-weighted and FLAIR images, with high signal intensity on diffusion-weighted imaging (DWI) and corresponding low signal on the apparent diffusion coefficient (ADC) map, indicating restricted diffusion (Figure 1). No vascular imaging (MRA/CTA) or perfusion studies (ASL/DSC) were performed due to early discharge at the family’s request. Therefore, while the imaging pattern is compatible with an acute ischemic or metabolic event, the absence of vascular data precludes definitive differentiation between a true vascular infarction and a mitochondrial stroke-like lesion.

**Figure 2:**
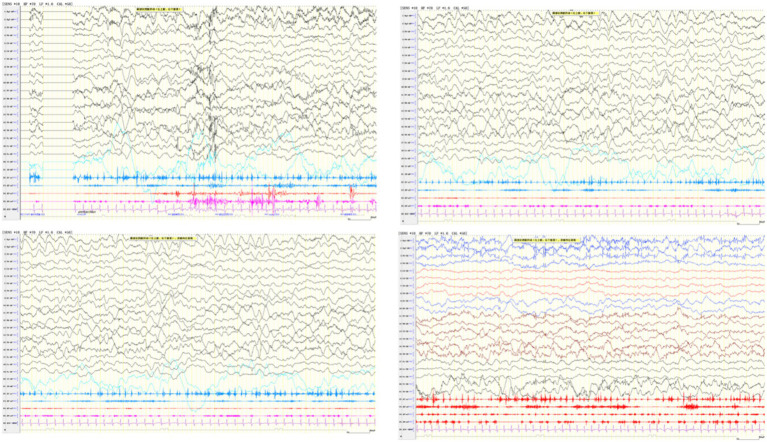
EEG demonstrates multispike–slow wave discharges, with or without transient EMG bursts in the left upper or right lower limb.

Electroencephalography (EEG) during medication-induced sleep showed diffuse slow waves (2–3 Hz) of variable amplitude with frequent multifocal spikes, sharp waves, and spike-slow wave discharges, predominantly in bilateral parietal, occipital, and posterior temporal regions. Electroclinical seizures were observed during monitoring, characterized by facial and limb twitching with leftward gaze deviation, accompanied by diffuse high-amplitude slow and spike–wave discharges. The seizures were terminated transiently after intravenous midazolam administration but recurred shortly afterward, consistent with epilepsia partialis continua (EPC) (Figure 2).

## 3 Materials and methods

### 3.1 Subjects

A 9-month-and-10-day-old girl (proband) with epilepsy were admitted at Department of Neurology, Children’s Hospital Affiliated to Shandong University. Trio-based whole exome sequencing (WES) was performed, via detailed method described in previous studies (10–16). This study was approved by the Institutional Research Ethics Committee of the Children’s Hospital Affiliated to Shandong University and written informed consent was obtained from the parents of the pedigree.

### 3.2 Genetic analysis

Peripheral blood samples were collected from the proband and her parents into EDTA-containing tubes for genomic DNA extraction using the QIAamp DNA Blood Midi Kit (Qiagen, Shanghai, China). DNA concentration and purity were assessed with a NanoDrop 2000 spectrophotometer (Thermo Fisher, United States).

Clinical exome sequencing was performed on the NovaSeq 6,000 platform (Illumina, United States) using the GenCap MedE006 capture kit (MyGenostics, Beijing, China). Detection of the large exon deletion in *NARS2* was performed by read-depth analysis of WES data. Candidate CNVs were prioritized based on significant reduction in coverage relative to the parents and internal controls. Sequencing achieved >95% coverage of target regions at ≥10 × depth, with an average depth exceeding 100×. Raw sequencing reads were aligned to the human reference genome (GRCh37/hg19: Although GRCh38 was released in 2013, we used GRCh37 due to its continued support in widely used annotation tools and databases, and to maintain consistency with previously published datasets) using Burrows–Wheeler Aligner (BWA, v0.7.10). Alignment files in SAM format were converted to BAM format for downstream processing. Post-alignment processing included local realignment with GATK’s RealignerTargetCreator and IndelRealigner, base quality score recalibration using GATK’s BaseRecalibrator, and variant calling with GATK HaplotypeCaller in “GENOTYPE_GIVEN_ALLELES” mode. Identified single nucleotide polymorphisms (SNPs) and insertions/deletions (indels) were filtered using GATK VariantFiltration (17).

Variants were annotated and functionally interpreted using ANNOVAR,^1^ and filtered against population databases including 1,000 Genomes, ExAC, gnomAD, ESP6500, and an in-house database, excluding variants with sub-allelic frequency >5%. Candidate variants were prioritized based on their relevance to the proband’s Human Phenotype Ontology (HPO) terms, a standardized nomenclature for systematically describing and classifying clinical phenotypes. The potential pathogenicity of novel variants was assessed using in silico prediction tools, including SIFT^2^ (v6.2.1), PolyPhen-2^3^ (v2.2.2), MutationTaster^4^ (v2021), SpliceAI^5^ (v1.3.1), and REVEL^6^ (v1.3). Additionally, previously reported variants were evaluated through the Human Gene Mutation Database (HGMD) and ClinVar. All variants were classified according to the 2015 American College of Medical Genetics and Genomics and the Association for Molecular Pathology guidelines (ACMG/AMP) guidelines, and suspected pathogenic variants were further confirmed by Sanger sequencing in the proband and her parents for segregation analysis (18). The functional impact of identified variants and their correlation with the patient’s clinical phenotype were evaluated using OMIM, dbSNP, Decipher, and relevant literature.

### 3.3 Bioinformatics analysis

Conservation analyses across a variety of species were conducted using ClustalX software. Structural modeling of both wild-type and mutant proteins was carried out utilizing the SWISSMODEL online tool,^7^ and PyMOL^8^ (version 2.5) was used for visualization and hydrogen bond analysis.

## 4 Results

### 4.1 Identification of NARS2 variants

**Figure 3:**
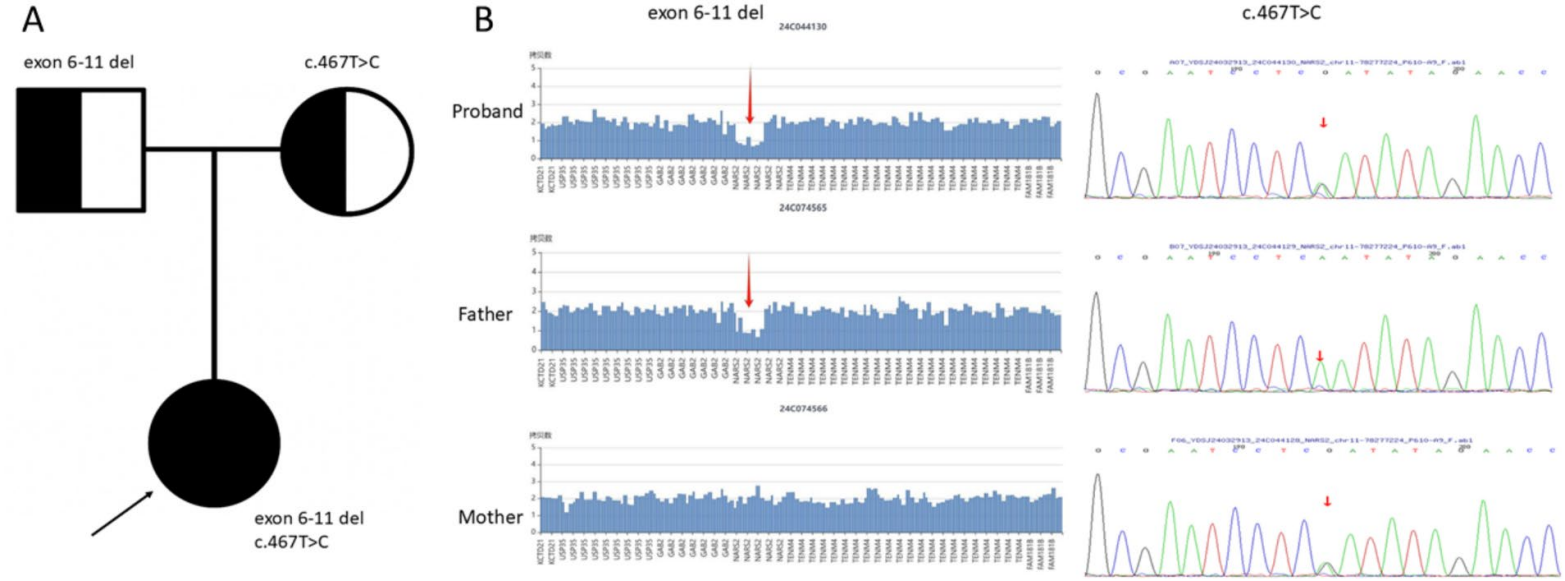
**(A)** The pedigree of this family. The proband affected with status seizures is indicated by black filled symbols and arrows. The parents who carried variants are displayed by symbols with black dots in the center. **(B)** Detection of the exon 6–11 heterozygous deletion and the missense variant c.467 T > C (p. Leu156Ser). The left panel shows the WES read-depth coverage plot from trio-based analysis, demonstrating markedly reduced coverage across exons 6–11 in the proband and half-reduced coverage in the father, consistent with a heterozygous multi-exon deletion. The right panel displays the Sanger sequencing chromatogram confirming the heterozygous missense variant c.467 T > C (p. Leu156Ser), inherited from the mother. Precise deletion breakpoints were not determined due to the resolution limits of short-read sequencing.

WES revealed compound heterozygous variants in the *NARS2* gene in the proband: a large deletion spanning exons 6–11 and a novel missense variant c.467 T > C (p. Leu156Ser). Based on exon structure, the exon 6–11 deletion is predicted to alter the protein length and likely disrupt the catalytic domain and is classified as likely pathogenic LP according to ACMG criteria (PVS1_Strong + PM2_Supporting). Segregation analysis showed that the deletion was inherited from the father, while the mother did not carry this deletion (Figure 3).

**Figure 4:**
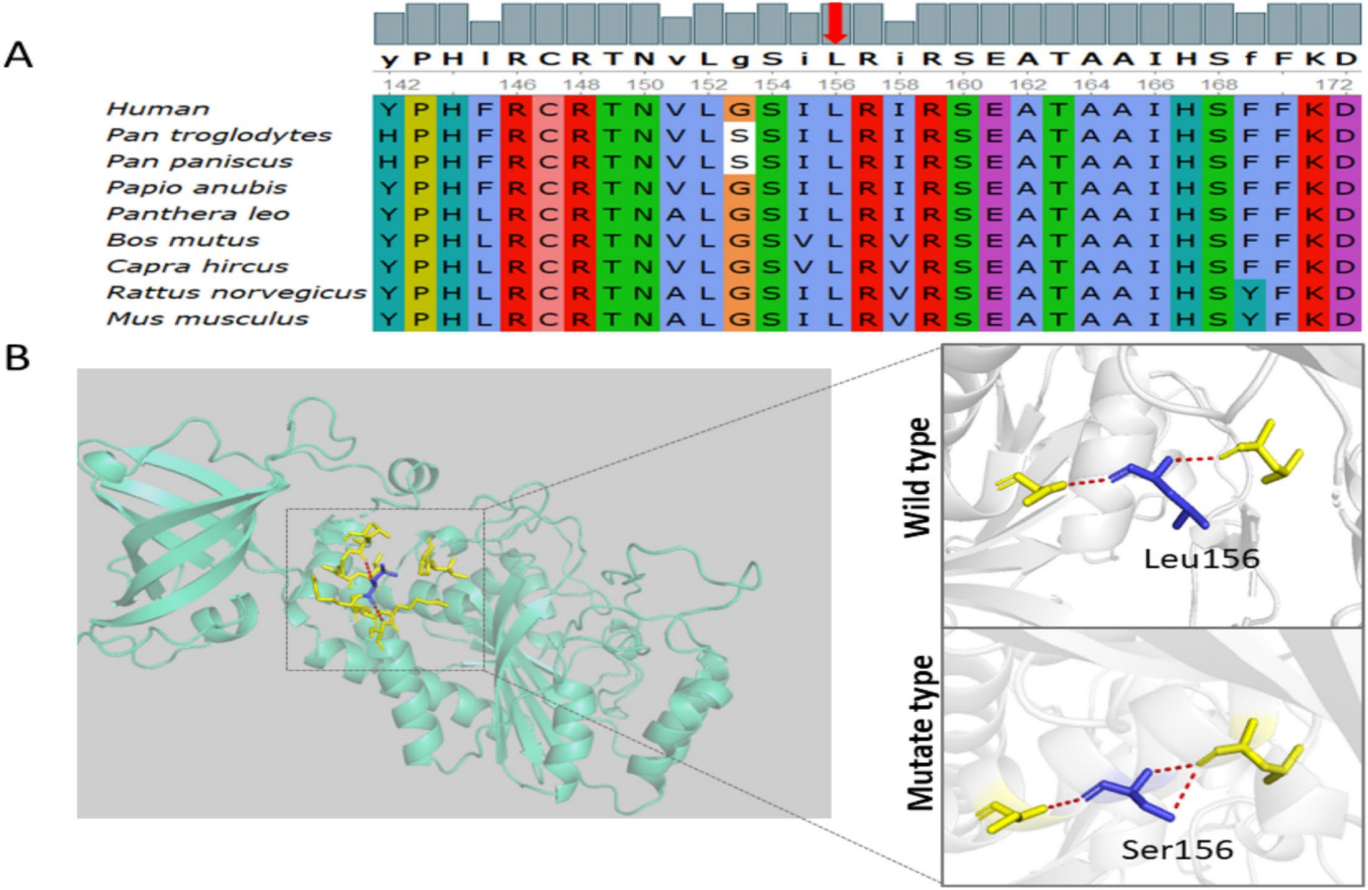
Evolutionary conservation and structural modeling of the *NARS2* p. Leu156Ser variant. **(A)** Multiple sequence alignment of *NARS2* across representative vertebrate species, ordered by evolutionary relatedness to humans. The conserved leucine residue at position 156 (highlighted with a red arrow) is centrally aligned in the figure, showing its strong evolutionary conservation across species. **(B)** Three-dimensional modeling of the NARS2 protein generated using SWISS-MODEL. The wild-type and mutant structures are shown on the right. The residue at position 156 is colored blue (Leu in wild type; Ser in mutant), and the surrounding residues involved in hydrogen bonding are shown in yellow. Red dashed lines represent hydrogen bonds. The p. Leu156Ser substitution alters the local hydrogen-bonding network, suggesting potential effects on protein stability or substrate interaction.

The c.467 T > C (p. Leu156Ser) variant results in substitution of leucine with serine at amino acid position 156. Leucine is a hydrophobic amino acid that contributes to the protein core, whereas serine is polar and can form hydrogen bonds and phosphorylation sites. This change from a hydrophobic to a hydrophilic residue is predicted to significantly disrupt local protein stability. Multiple sequence alignment demonstrated that the leucine residue at position 156 is highly conserved across diverse species, suggesting an important structural or functional role of this amino acid within the catalytic domain of mitochondrial asparaginyl-tRNA synthetase (Figure 4A). Three-dimensional protein modeling was performed using SWISSMODEL and PyMOL to compare the wild-type and mutant structures. The substitution of leucine by serine at position 156 resulted in altered local hydrogen bonding and disruption of the hydrophobic core surrounding the catalytic pocket (Figure 4B). Residue 156 resides within the catalytic domain of NARS2, critical for aminoacyl-tRNA synthetase activity; the substitution is predicted to impair formation of aminoacyl adenylate intermediates, potentially reducing enzymatic efficiency. According to ACMG guidelines, PM2_Supporting reflects absence from population databases, and PP3_Moderate is supported by in silico predictions (REVEL) indicating deleterious effects. ClinVar and literature searches revealed no prior reports of this variant. Sanger sequencing confirmed that the mother is heterozygous for the c.467 T > C variant, while the father carries the reference allele at this position. Together, these findings indicate that the proband’s compound heterozygous genotype can account for her clinical phenotype in an autosomal recessive inheritance pattern.

Taken together, the compound heterozygous variants—a truncating exon 6–11 deletion leading to loss of protein function and a missense substitution (p. Leu156Ser) predicted to potentially reduce enzymatic stability provide molecular evidence supporting the diagnosis of *NARS2*-related COXPD24. The identification of these two previously unreported variants expands the mutational spectrum of *NARS2* and underscores the importance of considering both copy number variants and subtle missense alterations in genetic testing for mitochondrial disorders.

### 4.2 Treatment and prognosis

Considering the proband’s clinical manifestations, frequent seizures, and genetic diagnosis of COXPD24, a multi-modal therapeutic approach was initiated. During hospitalization, she received oral lacosamide, perampanel, and clobazam, in combination with a ketogenic diet. Despite these interventions, seizures persisted intermittently. The family subsequently requested voluntary discharge, and at the time of discharge, occasional seizures were still observed. After discharge, no further follow-up visits were recorded, and seizure status was not systematically monitored thereafter.

### 4.3 Literature review

**Figure 5:**
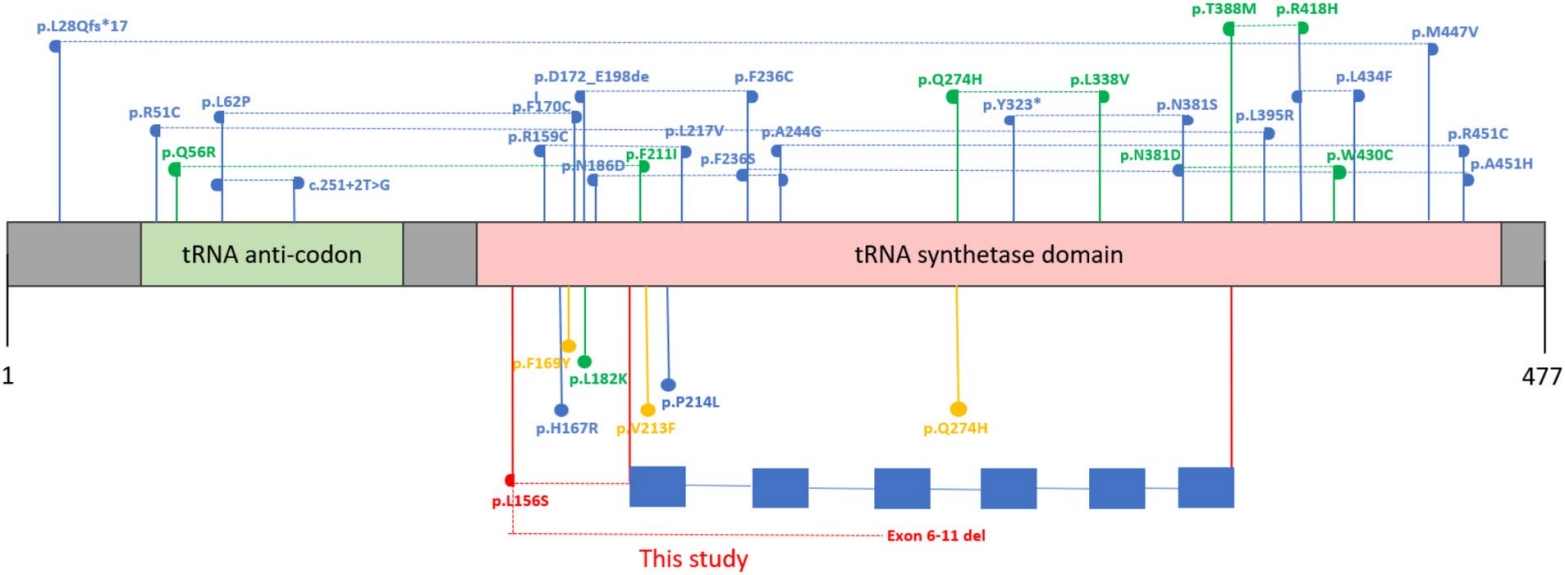
Distribution of reported and novel NARS2 variants and associated phenotypes. The NARS2 protein consists of an N-terminal OB-fold nucleic acid–binding domain (green) and a C-terminal aminoacyl-tRNA synthetase domain (red). All previously reported pathogenic variants are shown in blue, with homozygous variants represented as circles. Compound heterozygous variants are indicated by semicircles connected with dashed lines. The two novel variants identified in the present study—an exon 6–11 deletion and a missense variant (p. Leu156Ser)—are highlighted in red. Variants associated primarily with sensorineural hearing loss or hearing impairment are marked in orange, all previously reported COXPD24 cases were marked in green. The corresponding exon structure of the NARS2 gene is displayed below for clarity. The canonical transcript NM_024678.6 was used for gene and protein annotation.

A literature search of PubMed and Google Scholar was performed up to August 1, 2025 using the terms “*NARS2*” and “mitochondrial asparaginyl-tRNA synthetase 2.” Reported cases with confirmed *NARS2* variants and detailed clinical information were included (Table 1). To date, cerebral infarction has not been reported in any previously described *NARS2*-related cases, highlighting the potential expansion of the phenotypic spectrum observed in the present patient. We have reviewed all previously reported *NARS2* variants and analyzed their distribution across protein structural domains. Variants are mainly located in the N-terminal OB-fold nucleic acid–binding domain and the C-terminal aminoacyl-tRNA synthetase (aaRS) catalytic domain, with only one variant outside these regions. Our analysis did not reveal any clear correlation between the affected structural domain and clinical phenotype. To investigate the relationship between gene variant type and clinical phenotype, we classified reported cases into earlier-onset non-syndromic hearing loss (DFNB94/Hypoacusis) versus mitochondrial-related phenotypes (e.g., COXPD24, Leigh syndrome, Alpers syndrome). Statistical analysis showed that all earlier-onset hearing loss cases (*n* = 8) were associated with homozygous variants, whereas mitochondrial phenotypes included both homozygous (*n* = 8) and compound heterozygous variants (*n* = 22). A Chi-square test confirmed a statistically significant association between variant type (homozygous vs. compound heterozygous) and clinical phenotype (χ^2^ = 13.98, df = 1, *p* < 0.001), indicating that earlier-onset hearing loss is predominantly linked to homozygous missense variants, while more severe mitochondrial phenotypes tend to involve truncating or compound heterozygous variants. These findings suggest that variant type and zygosity, rather than structural domain location alone, may be the primary determinants of phenotypic severity, providing quantitative support for the observed genotype–phenotype correlations in NARS2-related disorders (Figure 5).

**Table 1:** Patients with mitochondrial disorders due to *NARS2* variants reported as of the end of 2025.

*NARS2* mutation	Published time	Diagnosis	Variant type	References	Case No.
c.822G > C; p. Q274H; c.1012C > G; p. Leu338Val	2015	COXPD24	Missense	Vanlander et al. (29)	1
c.822G > C; p. Q274H; c.1012C > G; p. Leu338Val	2015	COXPD24	Missense	Vanlander et al.	2
c.969 T > A; p. Tyr323* c.1142A > G; p. Asn381Ser	2015	Leigh Syndrome	Truncation Missense	Simon et al. (6)	3
c.969 T > A; p. Tyr323* c.1142A > G; p. Asn381Ser	2015	Leigh Syndrome	Truncation Missense	Simon et al.	4
c.637G > T; p. Val213Phe	2015	DFNB94	Missense	Simon et al.	5
c.637G > T; p. Val213Phe	2015	DFNB94	Missense	Simon et al.	6
c.637G > T; p. Val213Phe	2015	DFNB94	Missense	Simon et al.	7
c.637G > T; p. Val213Phe	2015	DFNB94	Missense	Simon et al.	8
c.641C > T; p. Pro214Leu	2015	Alpers syndrome	Missense	Sofou et al.	9
c.707 T > G; p. Phe236Cys c.594 +1G > A; p. Asp172_Glu198del	2017	Infantile-onset neurodegenerative disorder	Missense	Mizuguchi et al. (30)	10
c.707 T > G; p. Phe236Cys c.594 +1G > A; p. Asp172_Glu198del	2017	Infantile-onset neurodegenerative disorder	Missense	Mizuguchi et al.	11
c.151C > T; p. Arg51Cys c.1184 T > G; p. Leu395Arg	2017	Infantile-onset neurodegenerative disorder	Missense	Mizuguchi et al.	12
c.500A > G; p. His167Arg	2017	Infantile-onset neurodegenerative	Missense	Mizuguchi et al.	13
c.167A > G; p. Gln56Arg c.631 T > A; p. Phe211Ile	2018	COXPD24	Missense	Seaver et al. (31)	14
c.167A > G; p. Gln56Arg c.631 T > A; p. Phe211Ile	2018	COXPD24	Missense	Seaver et al.	15
c.731C > G; p. Ala244Gly c.1351C > T; p. Arg451Cys	2020	Leigh syndrome	Missense	Lee et al. (32)	16
c.270C > T; p. Asn90Asn	2020	Reversible COX deficiency	Synonymous	Palombo et al. (33)	17
c.641C > T; p. Pro214Leu	2021	Alpers syndrome	Missense	Sofou et al. (7)	18
c.641C > T; p. Pro214Leu	2021	Alpers/Leigh syndrome	Missense	Sofou et al.	19
c.545 T > A; p. Ile182Lys	2021	COXPD24	Missense	Vafaee-Shahi et al. (2)	20
c.545 T > A; p. Ile182Lys	2021	COXPD24	Missense	Vafaee-Shahi et al.	21
c.83_84del; p. Leu28Glnfs*17 c.1339A > G; p. Met447Val	2021	Focal seizures; status epilepticus	Truncation missense	Sterbova et al. (34)	22
c.1141A > G; p. Asn381Asp c.1290G > C; p. Trp430Cys	2022	COXPD24	Missense	Zhang et al. (4)	23
c.475C > T; p. Arg159Cys c.649 T > G; p. Leu217Val	2022	Epilepsy; neonatal diabetes syndrome	Missense	Yagasaki et al. (35)	24
c.475C > T; p. Arg159Cys c.649 T > G; p. Leu217Val	2022	Epilepsy; neonatal diabetes syndrome	Missense	Yagasaki et al.	25
c.1253G > A; p. Arg418His c.1300C > T; p. Leu434Phe	2022	Leigh syndrome	Missense	Yang et al. (36)	26
c.556 A > G; p. Asn186Asp c.731C > G; p. Ala244Gly	2022	Leigh syndrome	Missense	Tanaka et al. (37)	27
c.506 T > A; p. Phe169Tyr	2022	DFNB94	Missense	Al-Sharif et al. (8)	28
c.500 A > G; p. His167Arg	2022	Type 1 diabetes;	Missense	Cokyaman et al. (38)	29
c.185 T > C; p. Leu62Pro c.251 +2 T > G	2022	Refractory epilepsy;epilepsia partialis developmental delay	Splicing	Hu et al.	30
c.185 T > C; p. Leu62Pro c.509 T > G; p. Phe170Cys	2022	Refractory epilepsy; epilepsia partialis	Splicing	Hu et al. (9)	31
c.822G > C; p. Gln274His	2022	Hypoacusis	Missense	Ait-El-Mkadem et al. (39)	32
c.822G > C; p. Gln274His	2022	Hypoacusis; ataxia, tremor, spasticity	Missense	Ait-El-Mkadem et al.	33
c.822G > C; p. Gln274His	2022	Hypoacusis; ataxia, tremor, spasticity	Missense	Ait-El-Mkadem et al.	34
c.1352G > A; p. Arg451His c.707 T > C; p. Phe236Ser	2023	Epilepsy; developmental delay,	Missense	Yang et al. (20)	35
c.182C > T, c.446A > AG	2023	Developmental delay; intellectual disability; intellectual disability;	Missense	Josef Finsterer et al. (40)	36
c.1253G > A/p. Arg418His c.1163C > T/p. Thr388Met	2025	Focal seizures regression of motor development	Missense	Wu et al. (21)	37
c.1253G > A/p. Arg418His c.1163C > T/p. Thr388Met	2025	COXPD24	Missense	Wu et al.	38
exon 6–11 del c.467 T > C (p. Leu156Ser)	2025	COXPD24	Del Missense	Our study	39

DFNB, Deafness, Autosomal Recessive; COXPD24, Combined Oxidative Phosphorylation Deficiency 24.

## 5 Discussion

Mitochondria are central to cellular energy metabolism, and their functional integrity is critical for the normal operation of high-energy-demand organs such as the brain, heart, and skeletal muscle. *NARS2*, encoding mitochondrial asparaginyl-tRNA synthetase, plays an essential role in aminoacylation of mitochondrial tRNA, ensuring proper synthesis of proteins involved in oxidative phosphorylation. From an evolutionary perspective, NARS2 arose early during eukaryotic evolution and belongs to the highly conserved class of mitochondrial aminoacyl-tRNA synthetases. Comparative sequence analyses across vertebrate and invertebrate species demonstrate strong conservation of the catalytic core, particularly the ATP-binding and tRNA recognition domains, highlighting its essential role in maintaining mitochondrial translation fidelity. Similar to CARS2 and other mt-aaRS enzymes, this high degree of evolutionary conservation underscores the fundamental importance of NARS2 function in energy metabolism and helps explain why pathogenic variants can lead to profound multisystem phenotypes (6, 19).

Since its first identification as a disease-causing gene in 2015, biallelic *NARS2* variants have been associated with a broad spectrum of severe and highly heterogeneous clinical phenotypes. Biallelic pathogenic variants in *NARS2* cause COXPD24, a clinically heterogeneous disorder characterized by early-onset epilepsy, global developmental delay, abnormal muscle tone, progressive encephalopathy, sensorineural hearing loss, cardiomyopathy, and elevated lactate levels. The severity and spectrum of manifestations vary widely (2, 20). In the present case, cerebral infarction was identified on MRI following status epilepticus, representing a previously unreported manifestation of *NARS2*-related COXPD24. This finding expands the phenotypic spectrum of the disease to include focal metabolic ischemic injury, highlighting that mitochondrial dysfunction can compromise local cerebrovascular energy homeostasis. While it remains to be determined whether these lesions reflect true vascular occlusion or metabolic stroke-like events, their topography, diffusion characteristics, and temporal association with seizure activity suggest an energy crisis–driven neuronal injury rather than primary vascular pathology (9, 21). This observation parallels stroke-like episodes reported in other mitochondrial disorders, such as MELAS, and underscores the vulnerability of metabolically active brain regions to mitochondrial energy deficits (22, 23).

Equally important, early elevation of myocardial enzymes in this case carries major prognostic significance. Previous reports have noted persistent elevation of CK-MB and cTnI despite normal coronary angiography—a phenomenon of “biochemical–imaging dissociation,” characteristic of mitochondria-specific myocardial injury (24, 25). Mechanistically, cardiomyocytes appear doubly vulnerable to NARS2 deficiency: first, ATP depletion directly suppresses sarcoplasmic reticulum calcium reuptake, causing diastolic dysfunction; second, insufficient tRNA^Asn aminoacylation impairs synthesis of mitochondrial-encoded subunits of respiratory complex I, leading to excessive mitochondrial superoxide generation (26–28). Notably, enzyme abnormalities typically precede clinical heart failure by an average of 18 months and show a transient doubling within 24 h after seizure onset, indicating acute myocardial stress injury. Therefore, we recommend routine cardiac enzyme screening for all confirmed patients, particularly within 24 h following seizure episodes. Early detection of subclinical myocardial injury may provide a critical therapeutic window for initiating cardioprotective interventions and potentially reducing the risk of sudden cardiac death.

In the present study, we identified two novel compound heterozygous variants in *NARS2*, including a large exon 6–11 deletion and a missense variant c.467 T > C (p. Leu156Ser). Both variants have not been previously reported in the literature or public databases such as gnomAD or ClinVar, thus expanding the known mutational spectrum of *NARS2*. From a molecular perspective, large exon-level deletions are rarely reported, and traditional whole-exome sequencing may overlook such copy number variants, emphasizing the importance of incorporating CNV detection in molecular diagnostics. The novel missense variant p. Leu156Ser resides in the catalytic aminoacylation domain and is predicted to disrupt hydrophobic interactions crucial for tRNA charging, potentially impairing enzymatic function. Segregation analysis confirmed that the proband inherited each variant from a different parent, consistent with an autosomal recessive inheritance pattern. The combination of a loss-of-function deletion and a potentially hypomorphic missense variant may explain the severe early-onset phenotype while allowing survival to 9 months. The coexistence of a large exon 6–11 deletion, predicted to produce a truncated protein potentially resulting in loss of function, and a deleterious missense variant provides a strong molecular explanation for the severe phenotype observed in our patient. This finding further underscores the importance of comprehensive genetic analysis that integrates both sequence and copy number variation (CNV) detection to avoid missing clinically significant large-scale rearrangements. Expanding the *NARS2* mutation spectrum not only enhances understanding of its genotype–phenotype correlations but also provides valuable references for molecular diagnosis and genetic counseling in similar mitochondrial disorders.

Our comprehensive review and statistical analysis of all published *NARS2* variants revealed that almost all pathogenic changes are located within functional domains, including the N-terminal OB-fold nucleic acid–binding domain and the C-terminal aminoacyl-tRNA synthetase domain, with only one reported variant located outside these regions. Although no definitive correlation between structural domain and clinical phenotype has been established, certain trends emerged from this analysis. Notably, all previously reported DFNB94 cases with isolated sensorineural hearing loss involve homozygous missense variants, whereas severe mitochondrial phenotypes such as COXPD24 more frequently result from truncating or compound heterozygous variants. These findings suggest that variant type and zygosity, rather than domain localization alone, may play a predominant role in determining phenotypic severity. These observations suggest that variant type and zygosity, rather than domain location alone, may influence phenotypic severity. Although all reported patients carry biallelic pathogenic variants in *NARS2*, disease expression and severity vary widely, indicating incomplete penetrance. This variability may reflect multiple interacting factors. First, the residual enzymatic activity of mutant NARS2 proteins likely modulates the extent of mitochondrial translation impairment. Second, differences in tissue-specific energy demand contribute to the selective vulnerability of the brain, heart, and auditory system. Third, nuclear modifier genes involved in mitochondrial biogenesis and translation, as well as mitochondrial DNA haplogroups, may influence penetrance through compensatory or synergistic effects. Finally, environmental and epigenetic factors—such as infection, fever, or prolonged seizures—can trigger metabolic crises and accelerate disease progression. Together, these genetic and non-genetic determinants may account for the broad phenotypic heterogeneity observed among individuals with *NARS2*-related COXPD24.

Our findings provide new insights into *NARS2*-related mitochondrial disease, highlighting the importance of comprehensive genetic testing, early recognition of acute manifestations, and multidisciplinary management to improve outcomes in affected individuals. Although follow-up after discharge was limited due to the patient’s decision to discontinue treatment, the available clinical and molecular data provide valuable insights into the phenotypic and genetic spectrum of *NARS2*-related COXPD24. However, the limited follow-up duration and lack of functional assays restrict our ability to establish definitive genotype–phenotype correlations or confirm the proposed mechanisms. Future studies integrating functional validation, longitudinal clinical data, and larger patient cohorts are warranted to clarify the molecular basis of vascular and cardiac involvement.

In summary, this report highlights three key novelties: (1) the first documented case of cerebral infarction in *NARS2*-related COXPD24, expanding the neuroimaging and clinical phenotype; (2) identification of a large exon 6–11 deletion and a novel missense variant, enriching the mutational spectrum and emphasizing the need for CNV analysis; and (3) mechanistic insights suggesting that mitochondrial energy failure may precipitate focal metabolic stroke-like lesions, providing a conceptual framework for understanding acute neurovascular complications in mt-aaRS–related disorders. These findings have implications for both diagnosis and management, underscoring the importance of early recognition, comprehensive genetic testing, and multisystem monitoring in affected patients.

## Data Availability

The original contributions presented in the study are included in the article/supplementary material, further inquiries can be directed to the corresponding authors.

## Footnotes

1
http://annovar.openbioinformatics.org/en/latest/

2
https://sift.bii.a-star.edu.sg/

3
http://genetics.bwh.harvard.edu/pph2/

4
http://www.mutationtaster.org/

5
https://spliceailookup.broadinstitute.org/

6
https://sites.google.com/site/revelgenomics/

7
http://www.swissmodel.expasy.org

8
https://pymol.org/2/
